# Efficient RNA interference method during caste differentiation with hormone treatment in the termite *Reticulitermes speratus* (Isoptera: Rhinotermitidae)

**DOI:** 10.3389/finsc.2023.1188343

**Published:** 2023-05-09

**Authors:** Ryutaro Suzuki, Yudai Masuoka, Ryohei H. Suzuki, Kiyoto Maekawa

**Affiliations:** ^1^ Graduate School of Science and Engineering, University of Toyama, Toyama, Japan; ^2^ Ishikawa Insect Museum, Hakusan, Japan; ^3^ Institute of Agrobiological Sciences, National Agriculture and Food Research Organization, Tsukuba, Japan; ^4^ Research and Development Headquarters, Earth Corporation, Ltd. Ako, Japan; ^5^ Academic Assembly, University of Toyama, Toyama, Japan

**Keywords:** caste differentiation, RNAi, juvenile hormone, 20-hydroxyecdysone, ecdysone receptor gene

## Abstract

Unveiling the proximate mechanism of caste differentiation is crucial for understanding insect social evolution, and gene function analysis is an important tool in this endeavor. The RNA interference (RNAi) technique is useful in termites, but its knockdown effects may differ among species. One of the most important model species in the field of termite sociogenomics is *Reticulitermes speratus* Kolbe (Isoptera: Rhinotermitidae). Presoldier and worker differentiation of this species can be artificially induced by juvenile hormone and 20-hydroxyecdysone application, respectively. However, appropriate RNAi technique of genes expressed during caste differentiation has never been considered. To clarify this issue, first, we injected nine different volumes of nuclease-free water (NFW, 0–404.8 nL) into workers and found that survival and caste differentiation rates were strongly reduced by the application of the top three largest volumes. Second, we injected double-stranded (ds) RNA of *ecdysone receptor* homolog (*RsEcR*) (2.0 µg/151.8 nL NFW) into workers with hormone treatments. The expression levels of *RsEcR* were significantly reduced at 9 days after dsRNA injection. *RsEcR* RNAi strongly affected both molting events during presoldier and worker differentiation induced by hormone treatments. The present results highlight the need for caution regarding injection volumes for RNAi experiments using hormone treatments. We suggest that the injection of dsRNA solution (2 µg; approximately 100–200 nL) is suitable for RNAi experiments during caste differentiation induced by hormone application in *R. speratus*.

## 1 Introduction

The complex society of eusocial insects, such as bees, ants, and termites, is maintained by the division of labor among castes (1). Because the acquisition of castes may be an important event for social evolution in these insects, molecular developmental analyses have been performed to reveal the genetic determinants underlying caste differentiation and/or the regulatory mechanism for caste-specific phenotypic formation, especially in the Hymenoptera (ants, bees, and wasps) (2–4). Molecular evidence in other social insects, including termites, has been accumulated (5–8), and insect sociogenomics is receiving increased attention in related fields of research (9).

Termites are hemimetabolous eusocial insects that are phylogenetically distantly related to holometabolous hymenopteran taxa. Because termite caste differentiation is a molting process with some specific morphological changes, to reveal the regulatory mechanism of caste differentiation, we should focus on the developmental processes during a molt (10, 11). Gene function analysis using RNA interference (RNAi) have been shown to be useful in termites, and the RNAi technique has been applied in some species (reviewed by 12). However, RNAi-based knockdown analyses during caste differentiation have been conducted mostly for soldier differentiation (13–18) because soldier differentiation can be easily induced by juvenile hormone (JH) treatment. To understand the commonality and diversity of caste differentiation mechanisms in termites, however, we should prepare an appropriate technique for gene function analysis during other molts induced by hormone treatment. Moreover, to reveal the specific phenomenon during differentiation of each caste (e.g., unique hormonal regulation; 11), it is necessary to compare the functions of genes among each molt.

*Reticulitermes speratus* Kolbe (Isoptera: Rhinotermitidae) is an important species for understanding the regulatory mechanism of caste differentiation because the differentiation of all castes, including soldiers, workers, and reproductives, can be induced artificially only in this species. Namely, presoldier (intermediate stage of soldier) differentiation can be induced by JH application to workers (19). Meanwhile, worker molt can be induced by 20-hydroxyecdysone (20E) application to workers (20). Replacement reproductives (neotenics) can be induced by the isolation of nymphs from natal nests (21). Moreover, in this species, genome sequencing was completed, and the comprehensive transcriptome analysis among all castes and during all caste differentiations were performed (8, 22). Thus, the appropriate method of gene functional analysis should be established in *R. speratus*. However, RNAi-based functional analysis with hormone treatment has not been performed in *R. speratus*, although the presoldier differentiation and worker molts can be induced from the same developmental stage (old-age workers). Generally, in insects, RNAi efficiencies are suggested to be different among species, even in the same order (23). Consequently, based on the previous analyses performed in termites, an effective RNAi method during caste differentiation with hormone treatment should be verified in *R. speratus*. RNAi analysis with no hormone treatment [using 2 μg of double-stranded RNA (about 260 bp) of the JH receptor gene (*Methoprene-tolerant*)] was previously succeeded in this species (24), and thus we especially focused on the effects of the volumes of injection.

In this study, we intended to confirm the RNAi effects on caste differentiation induced by two hormones. Both JH and 20E hormones are crucial intrinsic factors for termite caste differentiation, always accompanied by the molting event. We therefore focused on the *ecdysone receptor* (*EcR*) gene, which is important for the molting event, including termite caste differentiation (25). Knockdown of this gene caused molting failure during soldier differentiation in *Zootermopsis nevadensis* (18). The effects of RNAi were confirmed based on the quantification of gene expression levels and phenotypic observations during caste differentiation. Based on these results, we propose a method for gene function analysis during caste differentiation *via* the treatment of *R. speratus* with both hormones.

## 2 Materials and methods

### 2.1 Termites

Mature *R. speratus* colonies were collected in Toyama Prefecture, Japan, in November 2018 and 2019. Pieces of logs housing the termites were brought to the laboratory and kept in plastic cases in constant darkness. Three colonies collected in 2018 (colonies A–C) were used to validate the injection volume. A colony collected in 2019 (colony D) was used for the RNAi analysis.

### 2.2 cDNA preparation

Total RNA for double-stranded RNA (dsRNA) synthesis was extracted from whole bodies of the 5th-6th stage workers (old-age workers) (five individuals in each sample) using ISOGEN II (Nippon Gene, Tokyo, Japan). Old-age workers were discriminated from other developmental stages based on the body size and antennal segments (26, 27). The extracted total RNA was purified using DNase treatment to remove genomic DNA. RNA purity and quantity were measured using a NanoVue spectrophotometer (GE Healthcare BioSciences, Tokyo, Japan). cDNA was synthesized from the purified RNA using a High-Capacity cDNA Reverse Transcription Kit (Applied Biosystems, Foster, CA, USA).

### 2.3 dsRNA synthesis

The ecdysone receptor homolog of *R. speratus* (*RsEcR*) was obtained from genome sequence data [*RsEcR* (gene ID: RS006194; 8)]. Using gene-specific primers (**Supplementary Table 1**; **Supplementary Figure 1**), the dsRNA of *RsEcR* was amplified. *RsEcR*-specific primers were adapted to the T7 promoter sequences. Gene-specific primers were designed using the Primer3 Plus software (28). To obtain template dsRNA, amplified *RsEcR* dsRNA was purified using a QIAquick gel extraction kit (Qiagen, Tokyo, Japan). In accordance with previous studies (14, 16, 24, 29), the *GFP* sequence for the control experiment was amplified using the GFP vector pQBI-poll I (Wako, Osaka, Japan). *GFP*-specific primers with T7 promoter sequences were newly designed using the Primer3 Plus (**Supplementary Table 1**; **Supplementary Figure 2**), because we intended to synthesize a shorter dsRNA (about 300 bp) (see Results). *RsEcR* and *GFP* dsRNA were synthesized using the MEGA script T7 Transcription Kit (Invitrogen, Carlsbad, CA, USA).

### 2.4 Validation of injection volume for RNAi

Nuclease-free water (NFW: solvent of dsRNA; 50.6, 101.2, 151.8, 202.4, 253.0, 303.6 354.2, and 404.8 nL, respectively) was injected into the lateral thorax of old-age workers of *R. speratus* (n = 60 per treatment) using a Nanoliter 2000 microinjector (World Precision Instruments, Sarasota, FL, USA) attached to a glass capillary. These individuals were kept in 65 mm petri dishes with 55 mm filter paper treated with 80 μg juvenile hormone III (JH III; Santa Cruz Biotechnology, Dallas, TX, USA) or 40 µg 20-hydroxyecdysone (20E; Sigma Aldrich, St. Louis, MO, USA) dissolved in 200 μL of acetone (20 individuals in each dish), in accordance with previous studies (19, 20, 30). All dishes were maintained in constant darkness at 25°C for 2 weeks and checked every 24 h to monitor the individuals. We calculated the rates of presoldier molt, worker molt and mortality. The rates of presoldier and worker molting individuals were percentages of those in survived individuals. The mortality were percentages of those in all the treated individuals. Statistical analysis was performed using two-way ANOVA followed by Tukey’s test with Mac statistical analysis ver. 3.0 (Esumi, Tokyo, Japan). Prior to the use of the ANOVA, we performed the Levene’s test on the variance equality using Mac statistical analysis ver. 3.0 (Esumi).

### 2.5 RNAi analysis and hormone treatment

RNAi was performed according to methods described in the previous study (24). *RsEcR* and *GFP* dsRNA (2 μg/151.8 nL) were injected into the side of the thorax of old-age workers of *R. speratus* (n = 50 per treatment) following the method described above. RNAi-treated individuals were kept in 65-mm petri dishes with 55-mm filter paper treated with 80 μg JH III (Santa Cruz Biotechnology) or 20 µg 20E (Sigma Aldrich) dissolved in 200 μL acetone (10 individuals in each dish). Before the RNAi experiment, workers collected from colony D were treated with different 20E concentrations (40, 20, 10, and 5 µg), because high mortality were observed when workers were treated with 40 µg 20E (see Results). These petri dishes were kept in an incubator at 25°C for 2 weeks to monitor the rates of molted presoldiers and workers. Induced and dead individuals were immediately removed from dishes and preserved in FAA solution (ethanol:formalin:acetic acid = 16:6:1) for 24 h and stored in 70% ethanol.

### 2.6 Real-time quantitative PCR (qPCR)

To check for the knockdown of gene expression, RNAi-treated individuals of *R. speratus* (n = 6 per treatment) were collected 6 and 9 days after RNAi treatment. Total RNA was extracted from the whole body of each individual using ISOGEN II (Nippon Gene, Tokyo, Japan). As described above, extracted RNA was used for cDNA synthesis, which was prepared for gene expression analysis of *RsEcR*. Relative quantification of transcripts was performed using PowerUp SYBR Green Master Mix (Applied Biosystems, Foster, CA, USA) and the QuantStudio 3 Real-Time PCR System (Applied Biosystems, Foster, CA, USA). Gene-specific primers for qPCR were designed using Primer3 Plus (**Supplementary Table 1**). According to the previous study (31), the suitability of six reference genes was evaluated using GeNorm (32) and NormFinder (33) software [*EF1-alpha* (accession No. AB602838; 34), *NADH-dh* (No. AB602837; 34), *beta-actin* (No. AB520714; 35), *glutathione S-transferase 1* (*GstD1*, gene ID: RS001168; 8), *ribosomal protein S18* (*RPS18*, ID: RS015150; 8), and *eukaryotic initiation factor 1A* (*eIF-1A*, ID: RS005199; 8)]. These were used as candidate reference genes in the respective termite species (24) and other insects, including *Drosophila melanogaster* and *Apis mellifera* (36, 37). Expression levels were calculated using biological replications (number of replications: n = 6). Statistical analysis was performed using the Mann-Whitney U test with Mac statistical analysis ver. 3.0 (Esumi).

### 2.7 Morphological observations

To evaluate the effects of *RsEcR* RNAi, we calculated the rates of individuals with gut purging (elimination of gut contents before the molt), presoldier induction and worker molting. The rates of gut-purged individuals were percentages of those in all the treated individuals. The rates of presoldier induction and worker molting individuals were percentages of those in gut-purged individuals. Statistical analysis was performed using Fisher’s test with Mac statistical analysis ver. 3.0 (Esumi).

## 3 Results and discussion

### 3.1 Effects of NFW injection on caste differentiation induced by hormone treatment

**Figure 1:**
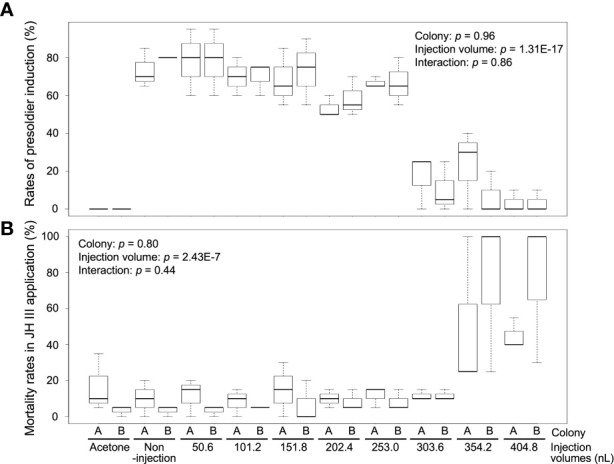
The rates of presoldier molt **(A)** and mortality **(B)** within 2 weeks after NFW (nuclease-free water) injection. The rates are calculated in each petri dish including 60 individuals (n = 3 dishes in each colony, **Supplementary Table 2**). NFW-injected workers were treated by juvenile hormone (JH) III application (80 µg per dish). Boxes and whiskers indicate the median, quartiles, and range. Statistical results of two-way ANOVA are shown in each graph (two-way ANOVA, *p* < 0.05). Both data are consistent with the use of parametric statistics by the Levene’s test [*p* = 0.8939 **(A)** and 0.8742 **(B)**].

To determine the optimal injection volume, we injected various volumes of NFW into the hormone-treated individuals. Presoldiers were strongly induced from workers (non-injection) by JH III treatment [73.3% ± 10.4% (colony A, mean ± SD) and 80.0 ± 0% (colony B)], and no presoldiers were observed in the control (acetone) treatment of both colonies (0%) (**Figure 1A**; **Supplementary Table 2**). The rates of presoldier molt in NFW-injected individuals varied among treatments with different injection volumes [two-way ANOVA, colony: *p* = 0.96, injection volume: *p* = 1.31E-17, interaction (colony *vs* injection volume): *p* = 0.86] (**Figure 1A**; **Supplementary Table 2**). In the treatments with 50.6–253.0 nL NFW injection, rates of presoldier molt were essentially similar to those of non-injection in both colonies (about 60–80%). However, presoldier molting rates were drastically dropped in the treatments with 303.6–404.8 nL injection (below 30%). Mortality were significantly different among treatments with NFW injection volumes after feeding JH III [two-way ANOVA, colony: *p* = 0.8, injection volume: *p* = 2.43E-7, interaction (colony *vs* injection volume): *p* = 0.44] (**Figure 1B**; **Supplementary Table 2**). High mortality was observed in treatments with large injection volumes.

**Figure 2:**
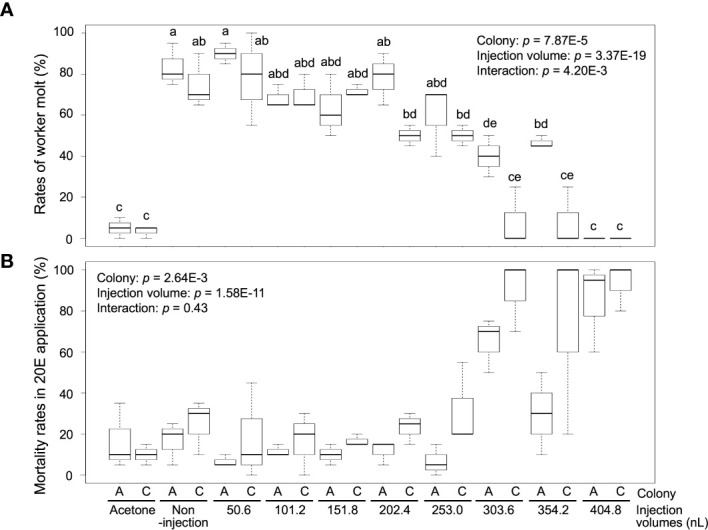
The rates of worker molt **(A)** and mortality **(B)** within 2 weeks after NFW (nuclease-free water) injection. The rates are calculated in each petri dish including 60 individuals (n = 3 dishes in each colony, **Supplementary Table 3**). NFW-injected workers were treated by 20-hydroxyecdysone (20E) application (40 µg per dish). Boxes and whiskers indicate the median, quartiles, and range. Statistical results of two-way ANOVA are shown in each graph. Different letters above the boxes indicate a significant difference (two-way ANOVA followed by Tukey’s test, *p* < 0.05). Both data are consistent with the use of parametric statistics by the Levene’s test [*p* = 0.8534 **(A)** and 0.9539 **(B)**].

Worker molt was strongly induced in workers (non-injection) by 20E treatment [83.3 ± 10.4% (colony A) and 75.0 ± 13.2% (colony C)], but almost never occurred in the control treatment [5.0% ± 5.0 (colony A) and 3.3 ± 2.9% (colony C)] (**Figure 2A**; **Supplementary Table 3**). The rates of worker molt were also affected by the different NFW injection volumes [two-way ANOVA, colony: *p* = 7.87E-5, injection volume: *p* = 3.37E-19, interaction (colony *vs* injection volume): *p* = 4.2E-3]. In the treatments with 50.6–253.0 nL injection, rates of worker molt were similar to those of non-injection in both colonies (about 60–80%). However, molting rates were significantly lower than those of non-injected individuals in the treatments with 303.6–404.8 nL injection in both colonies (below 40%). Mortality was significantly different among treatments with NFW injection volumes after feeding 20E [colony: *p* = 2.64E-3, injection volume: *p* = 1.58E-11, interaction (colony *vs* injection volume): *p* = 0.43] (**Figure 2B**; **Supplementary Table 3**). High mortality were observed in the treatments with large injection volumes, similar to JH III application.

Termite soldier differentiation requires high JH titers in the worker body (38, 39). Moreover, in insects, 20E titer levels generally increase before the larval molt (40). There is a possibility that the reduction in the molting rates is due to the dilution of hormone titer levels with the injected NFW (303.6–404.8 nL). Alternatively, the high mortality observed with large volumes of NFW injections may be due to some mechanical effects using pure water. To clarify this possibility, further injection analysis using a physiological saline solution should be performed. In the damp-wood termite *Hodotermopsis sjostedti*, RNAi-based knockdown was effectively induced by the injection of 1 µL dsRNA solution to the 7th instar treated with JH analog (15). We suggest that the proper (non-lethal) volumes of injection should be determined, especially considering the body size of the target individuals, because *H. sjostedti* 7th instars are much larger than the *R. speratus* workers used in this study. For RNAi-based knockdown with hormone treatment in *R. speratus*, injection volumes should be below 253 nL.

### 3.2 Effects of *EcR* RNAi on caste differentiation induced by hormone treatment

**Figure 3:**
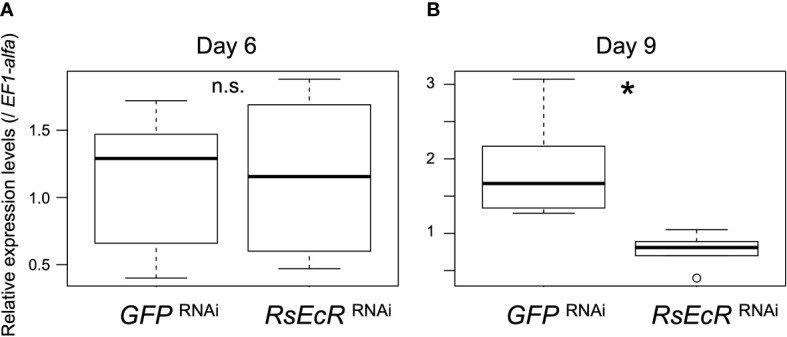
Expression levels of *RsEcR* (n = 6) 6 days **(A)** and 9 days **(B)** after RNAi treatment. Total RNA was extracted from the whole body of each individual, and six different individuals were used for each treatment. Boxes and whiskers indicate the median, quartiles, and range. An asterisk over the boxes indicates a significant difference (Mann-Whitney *U* test, *p* < 0.05). The term n.s. means not significant by statistical test.

A previous study showed that RNAi-based knockdown was effectively caused by injection of 2 µg dsRNA (about 260 bp) in *R. speratus* nymphs without hormone treatment (24). In *Drosophila* S2 cells, the length of dsRNA for effective RNAi was shown to be >211 bp (41). In this study, we prepared 2 µg dsRNA (about 300 bp) dissolved in 151.8 nL. According to the results described above, injection volumes should be reduced as small as possible (50.6 nL in this study). However, when we used injection volumes of 50.6 nL, a glass capillary was immediately clogged probably due to high viscosity. We then selected injection volumes of 151.8 nL, and injected *GFP* or *RsEcR* dsRNA solution (2.0 µg/151.8 nL) into *R. speratus* workers. *EF1-alpha* was selected as the most appropriate reference gene for real-time qPCR analyses (**Supplementary Table 4**). Gene expression levels of *RsEcR* were not affected by *RsEcR* RNAi 6 days after injection (day 6, **Figure 3A**; **Supplementary Table 5**). However, the expression levels of *RsEcR* were significantly decreased by *RsEcR* RNAi on day 9 (more than 50%) compared to the *GFP* control (**Figure 3B**; **Supplementary Table 5**). We did not observe any gross morphological and phenotypic changes in the RNAi-treated individuals without hormone treatments.

**Figure 4:**
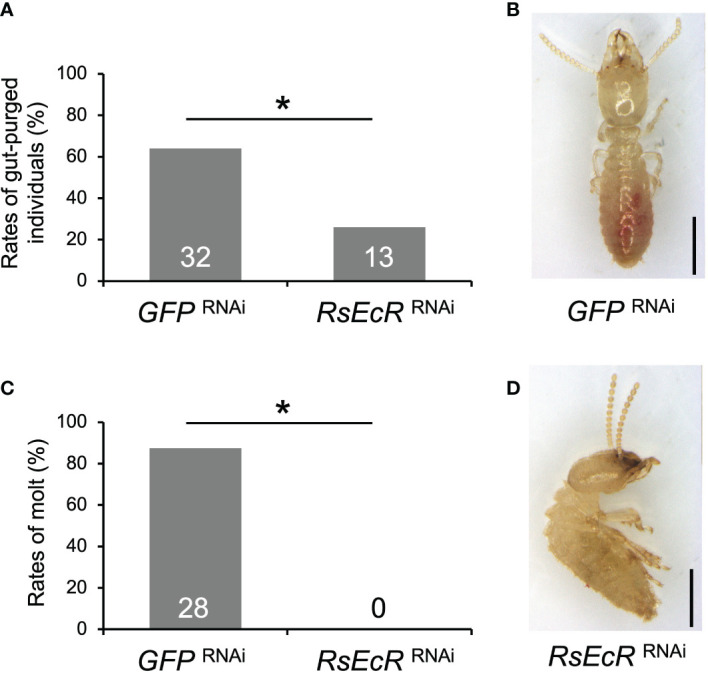
Phenotypic effects of *RsEcR* RNAi in workers treated by JH III application (80 μg per dish). Only one colony collected in 2019 (colony D) was used for the RNAi analysis. The rates of gut-purged **(A)** and molted individuals **(B)** within 2 weeks after RNAi treatment. The rates are calculated by the numbers of gut-purged individuals per 50 individuals examined **(A)**, and by the numbers of molted individuals per gut-purged individuals **(B)**. The numbers of individuals examined are indicated in each bar (**Supplementary Table 6**). An asterisk over the bars indicates a significant difference (Fisher’s test, *p* < 0.05). The typical phenotypes of the *GFP* RNAi-treated individual after the molt (**C**, normal presoldier) and the *RsEcR* RNAi-treated dead individual before the molt (**D**, dead old-age worker). Scale bar indicates 1 mm.

In *RsEcR* RNAi with JH treatment, the rates of gut-purged individuals (26%) were significantly lower than those in the *GFP* control (64%) (Fisher’s test, *p* < 0.05; **Figure 4A**; **Supplementary Table 6**). Furthermore, presoldiers emerged from most gut-purged individuals in the *GFP* control (87.5%; **Figures 4B, C**), but never from those in *RsEcR* RNAi treatment (0%; **Figure 4B**; **Supplementary Table 6**). All gut-purged individuals in the latter failed to molt (**Figure 4D**), as shown in *Z. nevadensis* (18).

**Figure 5:**
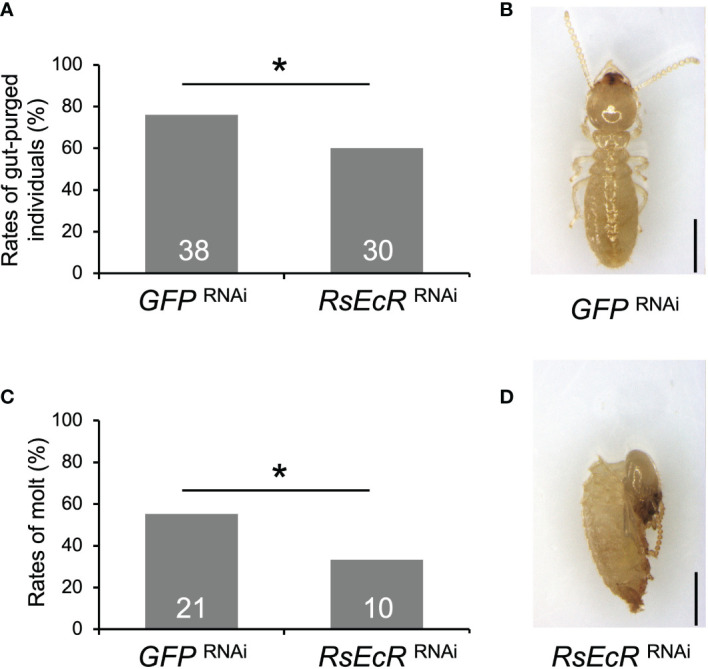
Phenotypic effects of *RsEcR* RNAi in workers treated by 20E application (20 μg per dish). Only one colony collected in 2019 (colony D) was used for the RNAi analysis. The rates of gut-purged **(A)** and molted individuals **(B)** within 2 weeks after RNAi treatment. The rates are calculated by the numbers of gut-purged individuals per 50 individuals examined **(A)**, and by the numbers of molted individuals per gut-purged individuals **(B)**. The numbers of individuals examined are indicated in each bar (**Supplementary Table 8**). An asterisk over the bars indicates a significant difference (Fisher’s test, *p* < 0.05). The typical phenotype of the *GFP* RNAi-treated individual after the molt (**C**, normal old-age worker), and the *RsEcR* RNAi-treated dead individual before the molt (**D**, dead old-age worker). Scale bar indicates 1 mm.

Before the experiment with *RsEcR* RNAi with 20E treatment, mortality of workers with 40, 20, 10, and 5 µg 20E were compared, because high mortality was observed in workers (colony D) treated with 40 µg 20E dissolved in 200 µL acetone. Although there were no statistical differences among treatments, the 20 µg 20E treatment tended to be higher rates of worker molt and lower levels of mortality (**Supplementary Figure 3**; **Supplementary Table 7**). Consequently, we decided to perform RNAi using 20 µg of 20E treatment. The rate of gut-purged individuals (60%) was significantly lower than that in the *GFP* control (76%) (Fisher’s test, *p* < 0.05; **Figure 5A**; **Supplementary Table 8**). *RsEcR* RNAi decreased the rates of worker molt (33.3%) compared to the control (55.3%) (**Figure 5B**; **Supplementary Table 8**), and most RNAi-treated individuals failed to molt (**Figures 5C, D**). These results indicate that the *RsEcR* RNAi assay performed here (injection of 2 µg dsRNA dissolved in 151.8 nL NFW) was successful in *R. speratus* workers with hormone treatments. Although the proper amount of dsRNA should be recognized for each gene, we suggest that the concentration obtained here can be used as the starting point for RNAi method during caste differentiation using hormone treatment in this species. According to the present study and previous works (15–18, 24), proper amount of dsRNA may be around 0.5–2 µg for the RNAi experiments in termites.

The relatively weaker effects of *RsEcR* RNAi treated with 20E (**Figure 5**), compared to those with JH III (**Figure 4**), may be due to the timing of knockdown effects caused by RNAi. Knockdown effects of RNAi could not be observed 6 days, but observed 9 days after dsRNA injection (**Figure 3**). Since the JH application induces the molting event to presoldiers, 20E-EcR action may be promoted after the increase of JH titer in workers treated with JH III. In contrast, 20E-EcR action may be immediately promoted by the 20E treatment in workers. Indeed, the initiation of molting event is occurred early in the 20E treatment, compared to the JH treatment (approximately 10-11 or 13-14 days after the treatment, respectively; 20). To clarify the possibility of the different timings of physiological action after each treatment, further expression analysis of hormone-related genes should be performed during caste differentiation.

## 4 Conclusions

We considered appropriate RNAi method during caste differentiation using hormone treatment in *R. speratus*. We suggest that it is necessary to regard not only dsRNA mass but also injection volumes using RNAi methods with hormone treatments. Using the method shown here, gene function analysis during caste differentiation can be performed effectively in *R. speratus*.

## Data availability statement

The original contributions presented in the study are included in the article/**Supplementary Materials**. Further inquiries can be directed to the corresponding author.

## Author contributions

RS, YM and KM designed this study. RS, RHS and KM collected samples. RS, YM and RHS performed experiments. RS, YM and KM analyzed the data. RS, YM and KM drafted the manuscript. All authors contributed to the article and approved the submitted version.
