# Supplementary material for: Efficient RNA interference method during caste differentiation with hormone treatment in the termite Reticulitermes speratus (Isoptera: Rhinotermitidae)
Source: Front Insect Sci. 2023 May 9;3:1188343. doi: 10.3389/finsc.2023.1188343 (PMC10926471; doi:10.3389/finsc.2023.1188343)
Supplement: Supplementary file 1 [file DataSheet_1.docx]

Supplementary Material

Efficient RNA interference method during caste differentiation with hormone treatment in the termite *Reticulitermes speratus* (Isoptera: Rhinotermitidae)

Ryutaro Suzuki, Yudai Masuoka, Ryohei H. Suzuki, Kiyoto Maekawa*

*** Correspondence:** Corresponding Author: kmaekawa@sci.u-toyama.ac.jp

# Supplementary Figures and Tables

## Supplementary Figures


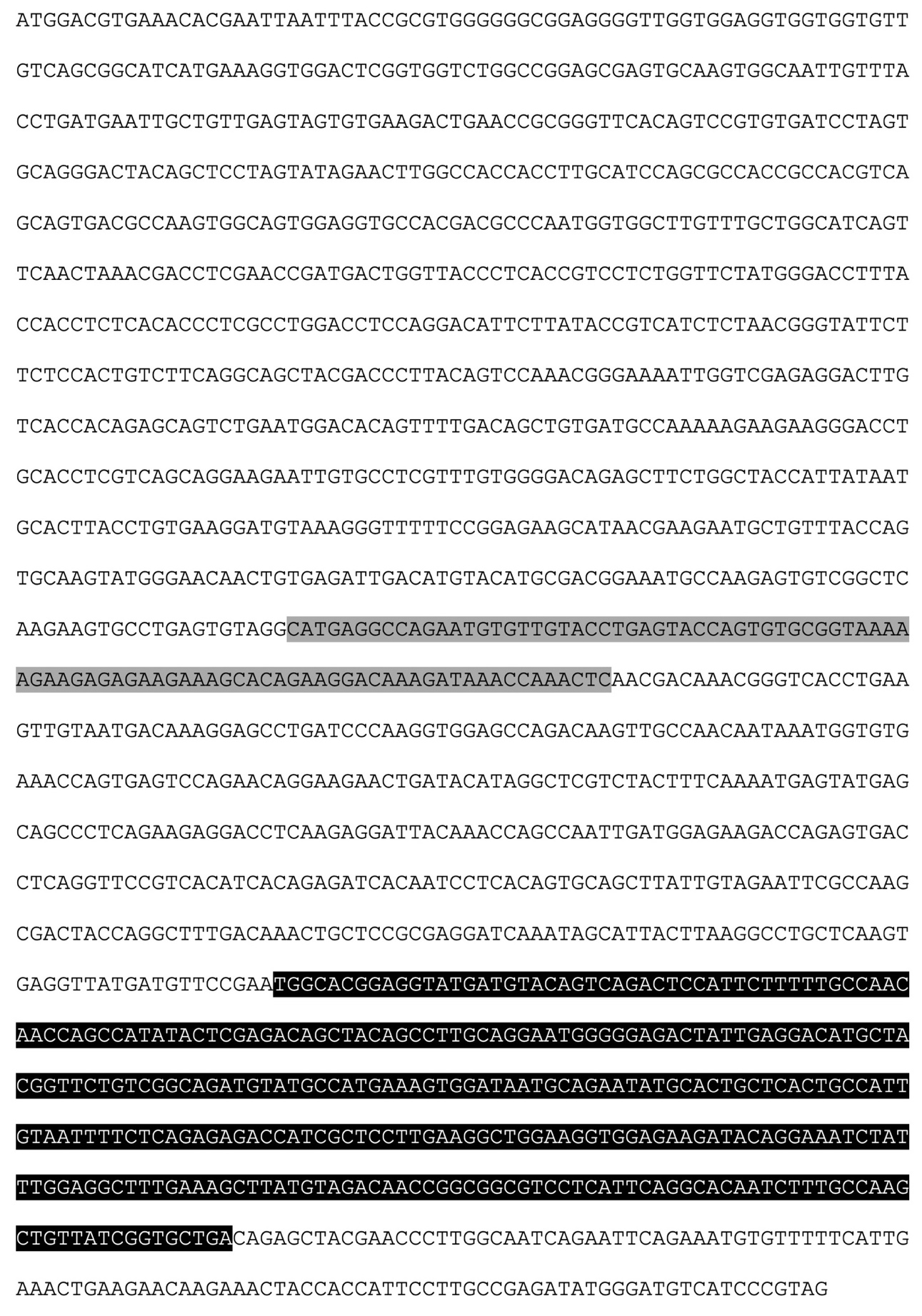


**Supplementary Figure 1.** DNA sequence of the ecdysone receptor gene of *Reticulitermes speratus* (*RsEcR*, ID: RS006194; Shigenobu et al., 2022). The region used for real-time qPCR is indicated as a gray box. White letters show the region used for dsRNA synthesis.

**Supplementary Figure 2.** DNA sequence of the *GFP* used for RNAi analysis. White letters show the region used for dsRNA synthesis (302 bp). Two underlined regions are primer positions used for dsRNA synthesis (706 bp) in previous studies (Masuoka et al., 2015; Saiki et al., 2015; Suzuki et al., 2019; Toga et al., 2012).


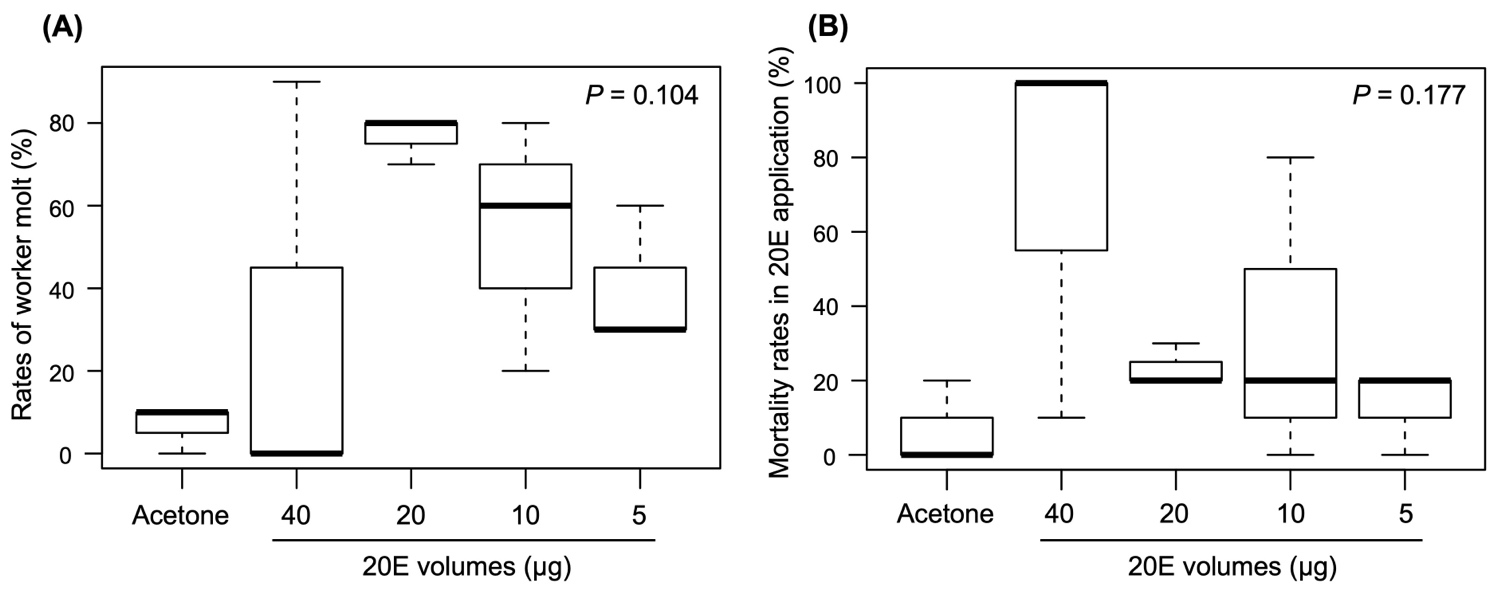


**Supplementary Figure 3.** Worker molt rates and mortality (n = 3) within 2 weeks after 20E application (A and B). These experiments were performed in workers collected from colony D, because high mortality were detected when workers were treated with 40 µg 20E dissolved in 200 μL acetone. Boxes and whiskers indicate the median, quartiles, and range. Statistical results of one-way ANOVA are shown in each graph (one-way ANOVA, *p* < 0.05).

## Supplementary Tables

| **Supplementary Table 1.** Primer sequences for dsRNA synthesis and real-time qPCR. | | |
| --- | --- | --- |
| Gene name | Forward or Reverse | Sequences (5' to 3') |
| *RsEcR* | Forward | T7 promoter + TGGCACGGAGGTATGATGTA |
| (for dsRNA synthesis) | Reverse | T7 promoter + GGGTTCGTAGCTCTGTCAGC |
| *GFP* | Forward | T7 promoter + GAAAGGGCAGATTGTGTGGA |
| (for dsRNA synthesis) | Reverse | T7 promoter + ACACGTGCTGAAGTCAAGTT |
| T7 promoter |  | TAATACGACTCACTATAGGG |
| *RsEcR* | Forward | CATGAGGCCAGAATGTGTTG |
| (for qPCR analysis) | Reverse | GACCCGTTTGTCGTTGAGTT |
| *EF1-alpha* | Forward | GGTGATGCGGCTATTGTTAACC |
| (for qPCR analysis) | Reverse | GTGGTGGGAATTCTGAGAAAGATT |
| *NADH-dh* | Forward | GCTGGGGGGGTTATTCATTCCAT |
| (for qPCR analysis) | Reverse | GGCATACCACAAAGGGCAAAA |
| *Beta-actin* | Forward | AGCGGGAAATCGTCCGTGAC |
| (for qPCR analysis) | Reverse | CAATGGTGATGACCTGCCCAT |
| *GstD1* | Forward | GCTGTTGGTGTGGATTTGAA |
| (for qPCR analysis) | Reverse | GTATGCTGCGGGTTCATCTT |
| *RSPS18* | Forward | ACTCTCAGCTCACATCCAGT |
| (for qPCR analysis) | Reverse | CCTCAGGCCCCAATAATGTC |
| *eIF-1A* | Forward | ATGGTAGGCTTGAAGCGATG |
| (for qPCR analysis) | Reverse | TTTGCATCCTGGTAGTCACG |

| **Supplementary Table 2.** Numbers of NFW-injected workers treated by JH III application (80 µg per dish). | | | | |
| --- | --- | --- | --- | --- |
| Category | Colony | Replication no. | No. of molted presoldiers | No. of dead individuals |
| Acetone | A | 1 | 0 | 1 |
| Acetone | A | 2 | 0 | 7 |
| Acetone | A | 3 | 0 | 2 |
| Acetone | B | 1 | 0 | 0 |
| Acetone | B | 2 | 0 | 1 |
| Acetone | B | 3 | 0 | 1 |
| Non injection | A | 1 | 17 | 0 |
| Non injection | A | 2 | 14 | 2 |
| Non injection | A | 3 | 13 | 4 |
| Non injection | B | 1 | 16 | 0 |
| Non injection | B | 2 | 16 | 1 |
| Non injection | B | 3 | 16 | 1 |
| 50.6 nL NFW | A | 1 | 19 | 0 |
| 50.6 nL NFW | A | 2 | 12 | 4 |
| 50.6 nL NFW | A | 3 | 16 | 3 |
| 50.6 nL NFW | B | 1 | 16 | 1 |
| 50.6 nL NFW | B | 2 | 19 | 0 |
| 50.6 nL NFW | B | 3 | 12 | 1 |
| 101.2 nL NFW | A | 1 | 14 | 3 |
| 101.2 nL NFW | A | 2 | 12 | 2 |
| 101.2 nL NFW | A | 3 | 16 | 0 |
| 101.2 nL NFW | B | 1 | 15 | 1 |
| 101.2 nL NFW | B | 2 | 15 | 1 |
| 101.2 nL NFW | B | 3 | 12 | 1 |
| 151.8 nL NFW | A | 1 | 13 | 3 |
| 151.8 nL NFW | A | 2 | 11 | 6 |
| 151.8 nL NFW | A | 3 | 17 | 0 |
| 151.8 nL NFW | B | 1 | 15 | 0 |
| 151.8 nL NFW | B | 2 | 18 | 0 |
| 151.8 nL NFW | B | 3 | 11 | 4 |
| 202.4 nL NFW | A | 1 | 10 | 1 |
| 202.4 nL NFW | A | 2 | 10 | 2 |
| 202.4 nL NFW | A | 3 | 12 | 3 |
| 202.4 nL NFW | B | 1 | 11 | 1 |
| 202.4 nL NFW | B | 2 | 10 | 1 |
| 202.4 nL NFW | B | 3 | 14 | 3 |
| 253.0 nL NFW | A | 1 | 13 | 3 |
| 253.0 nL NFW | A | 2 | 13 | 3 |
| 253.0 nL NFW | A | 3 | 14 | 1 |
| 253.0 nL NFW | B | 1 | 16 | 1 |
| 253.0 nL NFW | B | 2 | 11 | 1 |
| 253.0 nL NFW | B | 3 | 13 | 3 |
| 303.6 nL NFW | A | 1 | 5 | 2 |
| 303.6 nL NFW | A | 2 | 0 | 2 |
| 303.6 nL NFW | A | 3 | 5 | 3 |
| 303.6 nL NFW | B | 1 | 1 | 2 |
| 303.6 nL NFW | B | 2 | 0 | 2 |
| 303.6 nL NFW | B | 3 | 5 | 3 |
| 354.2 nL NFW | A | 1 | 6 | 5 |
| 354.2 nL NFW | A | 2 | 0 | 20 |
| 354.2 nL NFW | A | 3 | 8 | 5 |
| 354.2 nL NFW | B | 1 | 0 | 20 |
| 354.2 nL NFW | B | 2 | 0 | 20 |
| 354.2 nL NFW | B | 3 | 4 | 5 |
| 404.8 nL NFW | A | 1 | 2 | 8 |
| 404.8 nL NFW | A | 2 | 0 | 8 |
| 404.8 nL NFW | A | 3 | 0 | 11 |
| 404.8 nL NFW | B | 1 | 2 | 6 |
| 404.8 nL NFW | B | 2 | 0 | 20 |
| 404.8 nL NFW | B | 3 | 0 | 20 |

| **Supplementary Table 3.** Numbers of NFW-injected workers treated by 20E application (40 µg per dish). | | | | |
| --- | --- | --- | --- | --- |
| Category | Colony | Replication no. | No. of molted workers | No. of dead individuals |
| Acetone | A | 1 | 1 | 1 |
| Acetone | A | 2 | 0 | 7 |
| Acetone | A | 3 | 2 | 2 |
| Acetone | C | 1 | 1 | 2 |
| Acetone | C | 2 | 1 | 1 |
| Acetone | C | 3 | 0 | 3 |
| Non injection | A | 1 | 15 | 5 |
| Non injection | A | 2 | 19 | 1 |
| Non injection | A | 3 | 16 | 4 |
| Non injection | C | 1 | 18 | 2 |
| Non injection | C | 2 | 13 | 7 |
| Non injection | C | 3 | 14 | 6 |
| 50.6 nL NFW | A | 1 | 18 | 1 |
| 50.6 nL NFW | A | 2 | 19 | 1 |
| 50.6 nL NFW | A | 3 | 17 | 2 |
| 50.6 nL NFW | C | 1 | 20 | 0 |
| 50.6 nL NFW | C | 2 | 11 | 9 |
| 50.6 nL NFW | C | 3 | 16 | 2 |
| 101.2 nL NFW | A | 1 | 15 | 3 |
| 101.2 nL NFW | A | 2 | 13 | 2 |
| 101.2 nL NFW | A | 3 | 13 | 2 |
| 101.2 nL NFW | C | 1 | 13 | 6 |
| 101.2 nL NFW | C | 2 | 16 | 0 |
| 101.2 nL NFW | C | 3 | 13 | 4 |
| 151.8 nL NFW | A | 1 | 16 | 2 |
| 151.8 nL NFW | A | 2 | 12 | 1 |
| 151.8 nL NFW | A | 3 | 10 | 3 |
| 151.8 nL NFW | C | 1 | 14 | 3 |
| 151.8 nL NFW | C | 2 | 14 | 3 |
| 151.8 nL NFW | C | 3 | 15 | 4 |
| 202.4 nL NFW | A | 1 | 16 | 3 |
| 202.4 nL NFW | A | 2 | 13 | 3 |
| 202.4 nL NFW | A | 3 | 18 | 1 |
| 202.4 nL NFW | C | 1 | 10 | 5 |
| 202.4 nL NFW | C | 2 | 9 | 6 |
| 202.4 nL NFW | C | 3 | 11 | 3 |
| 253.0 nL NFW | A | 1 | 14 | 0 |
| 253.0 nL NFW | A | 2 | 14 | 1 |
| 253.0 nL NFW | A | 3 | 8 | 3 |
| 253.0 nL NFW | C | 1 | 9 | 11 |
| 253.0 nL NFW | C | 2 | 10 | 4 |
| 253.0 nL NFW | C | 3 | 11 | 4 |
| 303.6 nL NFW | A | 1 | 8 | 15 |
| 303.6 nL NFW | A | 2 | 6 | 14 |
| 303.6 nL NFW | A | 3 | 10 | 10 |
| 303.6 nL NFW | C | 1 | 5 | 14 |
| 303.6 nL NFW | C | 2 | 0 | 20 |
| 303.6 nL NFW | C | 3 | 0 | 20 |
| 354.2 nL NFW | A | 1 | 9 | 10 |
| 354.2 nL NFW | A | 2 | 9 | 6 |
| 354.2 nL NFW | A | 3 | 10 | 2 |
| 354.2 nL NFW | C | 1 | 0 | 20 |
| 354.2 nL NFW | C | 2 | 5 | 4 |
| 354.2 nL NFW | C | 3 | 0 | 20 |
| 404.8 nL NFW | A | 1 | 0 | 12 |
| 404.8 nL NFW | A | 2 | 0 | 19 |
| 404.8 nL NFW | A | 3 | 0 | 20 |
| 404.8 nL NFW | C | 1 | 0 | 16 |
| 404.8 nL NFW | C | 2 | 0 | 20 |
| 404.8 nL NFW | C | 3 | 0 | 20 |

| **Supplementary Table 4.** Stability values of reference genes using GeNorm and NormFinder. | | |
| --- | --- | --- |
| Gene name | Stability value | |
|  | GeNorm | NormFinder |
| *EF1-alpha** | 0.449 | 0.071 |
| *NADH-dh* | 0.489 | 0.087 |
| *Beta-actin* | 0.667 | 0.404 |
| *GstD1* | 0.530 | 0.087 |
| *RPS18* | 0.529 | 0.250 |
| *eIF-1A* | 1.119 | 0.761 |
| **EF1-alpha* was selected due to the lowest stability values. | | |

| **Supplementary Table 5.** Relative expression levels calculated from standard curve in each gene. | | | | |
| --- | --- | --- | --- | --- |
| Treatment | Day | Replication No. | *EcR* | *EF1* |
| *GFP* RNAi | 6 | 1 | 0.037127126 | 0.092650354 |
| *GFP* RNAi | 6 | 2 | 0.080078632 | 0.059932336 |
| *GFP* RNAi | 6 | 3 | 0.028816642 | 0.023267481 |
| *GFP* RNAi | 6 | 4 | 0.019187892 | 0.028989265 |
| *GFP* RNAi | 6 | 5 | 0.09351892 | 0.054353762 |
| *GFP* RNAi | 6 | 6 | 0.121621259 | 0.082787365 |
| *GFP* RNAi | 9 | 1 | 0.058838371 | 0.046162382 |
| *GFP* RNAi | 9 | 2 | 0.034877598 | 0.016090855 |
| *GFP* RNAi | 9 | 3 | 0.061886586 | 0.044449348 |
| *GFP* RNAi | 9 | 4 | 0.135358736 | 0.04405982 |
| *GFP* RNAi | 9 | 5 | 0.052803989 | 0.039363462 |
| *GFP* RNAi | 9 | 6 | 0.170013577 | 0.087079421 |
| *EcR* RNAi | 6 | 1 | 0.064707272 | 0.107332736 |
| *EcR* RNAi | 6 | 2 | 0.032688994 | 0.069109216 |
| *EcR* RNAi | 6 | 3 | 0.29534477 | 0.197858855 |
| *EcR* RNAi | 6 | 4 | 0.480917096 | 0.255791962 |
| *EcR* RNAi | 6 | 5 | 0.086315461 | 0.105647117 |
| *EcR* RNAi | 6 | 6 | 0.192673311 | 0.114216842 |
| *EcR* RNAi | 9 | 1 | 0.102680646 | 0.253659427 |
| *EcR* RNAi | 9 | 2 | 0.121823505 | 0.174805626 |
| *EcR* RNAi | 9 | 3 | 0.125763908 | 0.140542254 |
| *EcR* RNAi | 9 | 4 | 0.213981748 | 0.245484605 |
| *EcR* RNAi | 9 | 5 | 0.15552178 | 0.147451565 |
| *EcR* RNAi | 9 | 6 | 0.083518803 | 0.111695617 |

| **Supplementary Table 6.** Numbers of individuals after JH III application (80 µg per dish). | | | | |
| --- | --- | --- | --- | --- |
| Treatment | No. of individuals examined | No. of gut-purged individuals | No. of molted presoldiers | No. of dead individuals |
| *GFP* RNAi | 50 | 32 | 28 | 2 |
| *EcR* RNAi | 50 | 13 | 0 | 15 |

| **Supplementary Table 7.** Numbers of individuals after 20E application. | | | |
| --- | --- | --- | --- |
| Category | Replication no. | No. of molted workers | No. of dead individuals |
| Acetone | 1 | 0 | 0 |
| Acetone | 2 | 1 | 2 |
| Acetone | 3 | 1 | 0 |
| 40 µg per dish | 1 | 0 | 10 |
| 40 µg per dish | 2 | 9 | 1 |
| 40 µg per dish | 3 | 0 | 10 |
| 20 µg per dish | 1 | 8 | 2 |
| 20 µg per dish | 2 | 7 | 3 |
| 20 µg per dish | 3 | 8 | 2 |
| 10 µg per dish | 1 | 2 | 8 |
| 10 µg per dish | 2 | 8 | 2 |
| 10 µg per dish | 3 | 6 | 0 |
| 5 µg per dish | 1 | 3 | 0 |
| 5 µg per dish | 2 | 6 | 2 |
| 5 µg per dish | 3 | 3 | 2 |

| **Supplementary Table 8.** Numbers of individuals after 20E application (20 µg per dish). | | | | |
| --- | --- | --- | --- | --- |
| Treatment | No. of individuals examined | No. of gut-purged individuals | No. of molted presoldiers | No. of dead individuals |
| *GFP* RNAi | 50 | 38 | 21 | 25 |
| *EcR* RNAi | 50 | 30 | 10 | 23 |
